# Hydration studies on the archaeal protein Sso7d using NMR measurements and MD simulations

**DOI:** 10.1186/1472-6807-11-44

**Published:** 2011-10-21

**Authors:** Andrea Bernini, Ottavia Spiga, Roberto Consonni, Ivana Arosio, Paola Fusi, Simone Cirri, Annamaria Guagliardi, Neri Niccolai

**Affiliations:** 1 Dipartimento di Biotecnologie, Università degli Studi di Siena, via Fiorentina 1, Siena, Italy; 2 ISMAC Lab. NMR, CNR, via Bassini 15, Milano, Italy; 3 Dipartimento di Biotecnologie e Bioscienze, Università di Milano-Bicocca, P.zza della Scienza 2, Milano, Italy; 4 Dipartimento di Biologia Strutturale e Funzionale, Università Federico II, Via Cinthia 4, Napoli, Italy

## Abstract

**Background:**

How proteins approach surrounding molecules is fundamental to our understanding of the specific interactions that occur at the surface of proteins. The enhanced surface accessibility of small molecules such as organic solvents and paramagnetic probes to protein binding sites has been observed; however, the molecular basis of this finding has not been fully established. Recently, it has been suggested that hydration dynamics play a predominant role in controlling the distribution of hot spots on surface of proteins.

**Results:**

In the present study, the hydration of the archaeal multifunctional protein Sso7d from *Solfolobus solfataricus *was investigated using a combination of computational and experimental data derived from molecular dynamics simulations and ePHOGSY NMR spectroscopy.

**Conclusions:**

We obtained a convergent protein hydration landscape that indicated how the shape and stability of the Sso7d hydration shell could modulate the function of the protein. The DNA binding domain overlaps with the protein region involved in chaperon activity and this domain is hydrated only in a very small central region. This localized hydration seems to favor intermolecular approaches from a large variety of ligands. Conversely, high water density was found in surface regions of the protein where the ATP binding site is located, suggesting that surface water molecules play a role in protecting the protein from unspecific interactions.

## Background

It is very unlikely that proteins interact randomly with their molecular environment. Water, the most ubiquitous and abundant molecule of life, certainly plays a major role in controlling intermolecular interactions among biomolecules. To understand the interactions in specific protein surface regions that trigger biological functions requires an accurate delineation of protein hydration dynamics.

Protein hydration at the atomic level can be investigated using a variety of independent techniques such as molecular dynamics (MD) simulations [1-4], high resolution X-ray crystallography [5,6] and NMR spectroscopy. Nuclear magnetic relaxation dispersion (MRD) studies seem to be particularly suited to determining the number of water molecules that are tightly bound to proteins [7,8], but cannot provide information at atomic resolution per se. In principle, a detailed spatial distribution of water molecules that exhibit long residence times can be defined from hydration NMR experiments based on cross-relaxation [9], by analyzing the water-protein Overhauser effect (NOE_wp_) arising from selective water excitation. Pulsed field gradients, generated by standard hardware, are included in ePHOGSY-type sequences [10] limiting typical artifacts due to selective pulses [11]. General developments of high resolution hydration NMR experiments have been critically reviewed [12] and signals observed in ePHOGSY-type spectra have been predicted also from chemical exchange or relayed Overhauser effects. Indeed, as suggested in the pioneering work of Wüthrich and co-workers [13], relayed water-protein Overhauser effects (NOE_(wp)_s) cannot be measured separately from NOE_wp_s [9]. The NOE_(wp)_s expected from all the amino acids bearing exchangeable hydrogens on their side chains are also propagated to hydrogens that are spatially close to the exchangeable side-chain hydrogen atoms. Because the amino acid side chains that bear the exchangeable hydrogen atoms are also the ones which are most frequently involved in the binding of water molecules [14], the interpretation of ePHOGSY signals in terms of protein hydration seems to be limited to only very few cases [9,15]. It has been suggested [16] that a partial solution to the latter problem could be to compare the hydration patterns obtained from NMR studies and from MD simulations. A convergence of the computationally and experimentally derived hydration data could provide mutual validation.

MD simulations have indicated that strong hydration sites are not found in surface regions where protein hot spots are present [1]. This result is of primary relevance to predictions of the functional properties of protein surfaces. ePHOGSY NMR studies have experimentally confirmed the absence of strong hydration sites at protein active sites [17]. Furthermore, as recently reviewed [18], a tight correlation exists between the absence of MD derived high water density sites and surface regions highly accessible to paramagnetic probes [16,19].

The 63 aminoacids long DNA binding protein, Sso7d, from the extreme thermophilic crenarchaeon *Sulfolobus solfataricus *supports multiple and structurally well-defined activities [20,21] making it a good model protein for analyzing the relationship between solvent dynamics, surface accessibility and biological function. Because Sso7d binds different substrates, such as nucleic acids, ATP and misfolded/unfolded proteins [22,23], as a model, it offers a unique opportunity to investigate how the different ligand molecules can adopt their own approach to bind to their targets on the protein surface. Sso7d surface accessibility has previously been studied using paramagnetic probes that preferentially accessed the specific surface regions where interactions with DNA and misfolded/unfolded proteins occurred [24]. The finding that some surface-exposed regions of Sso7d were equally inaccessible to TEMPOL and Gd(III)(DTPA-BMA) has been ascribed to the presence of tightly bound water molecules [24].

The hydration of Sso7d and the homologous protein Sac7d from *Sulfolobus acidocaldarius *has been analyzed from crystal structures of DNA-protein complexes [25,26]. In these complexes, four water molecules that were differently arranged in a diamond shape were found at the interface between Sso7d/Sac7d and the bound DNA. The four water molecules were apparently acting as a molecular filler to optimize protein-DNA binding. A series of conserved water molecules, including those that were involved in the Sso7d ATPase activity, were also observed. The structural stability of Sso7d [24], prevented major conformational changes of the protein backbone within the MD simulation timescale and allowed protein hydration investigations to be carried out using the MD Hydration Site (MDHS) approach [1,4]. In the present work, Sso7d hydration was studied using MD simulations and ePHOGSY NMR spectroscopy to identify correlations between solvent dynamics and the functional features encoded in the protein surface.

## Results

### Structural alignment

**Figure 1:**
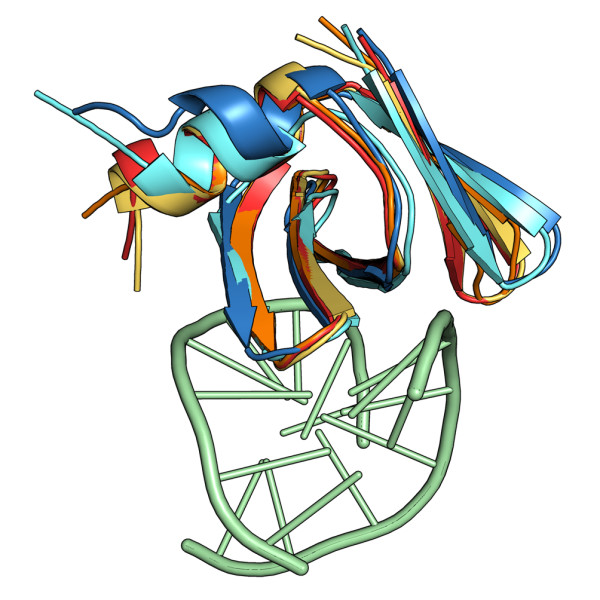
**Sso7d structure dataset**. Superposition of three X-ray structures of Sso7d with a bound DNA duplex, yellow [PDB:1BNZ], orange [PDB:1C8C], red [PDB:1BF4], and of two NMR structures for the free protein, cyan [PDB:1JIC], and blue [PDB:1SSO]. The DNA, shown in green, is from PDB:1C8C.

Two solution structures of free Sso7d [27,28] and three crystal structures of the protein complexed with different DNA fragments [25,26] are available in the Protein Data Bank [29]. As shown in Figure 1, the complexed forms of Sso7d are very similar, particularly in the protein/DNA binding region. High similarity between the two NMR structures is also apparent, confirming that only small differences in orientation and packing of backbone and side chain groups are allowed in the Sso7d crystal structures. The mean global RMSD calculated for the backbone atoms of the five structures, excluding four disordered residues at the C-terminus and Met1, was only 0.15 nm. However, to minimize artifacts due to initial local conformations, one solution structure and one crystal structure have been used as the reference structures for the two MD simulations (see next sub-section).

### Molecular Dynamics simulation

**Figure 2:**
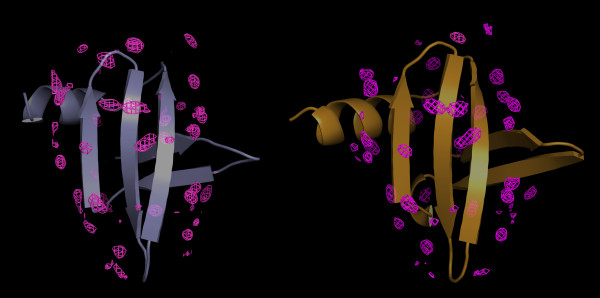
**Molecular Dynamics Hydration Site maps**. MDHS maps obtained from MD simulations carried out on the representative structures for solution NMR (left) [PDB:1JIC] and X-ray crystallography (right) [PDB:1C8C]. Protein structures are shown as transparent ribbons; the MD hydration sites are shown in magenta; the plotted mesh iso-surfaces were at level 2.8.

**Figure 3:**
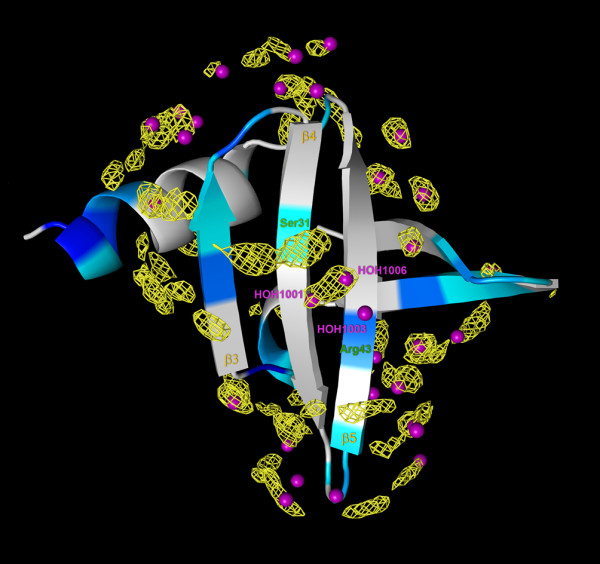
**Distribution of MDHS, conserved water molecules and ePHOGSY NMR signals in the Sso7d structure**. Ribbon representation of Sso7d [PDB:1C8C] showing the ePHOGSY signal intensity, the darker the blue, the stronger the signal; grey indicates regions where no signal was detected. The MDHS are shown in yellow; the plotted mesh iso-surfaces were at level 2.0. The water molecules that were found to be conserved in the available crystal structures are shown as purple spheres.

To minimize biased contributions from the Sso7d reference structures to the dynamics of the surface water molecules, two 100 ns MD simulations in explicit water were carried out using one crystal structure [PDB: 1C8C] and one solution structure [PDB: 1JIC]. From each MD trajectory the solvent density map was computed and the maxima of the MDHS [1] are given in Figure 2. Quantitative correlation of the two density maps was computed with a coefficient of 0.71 at the density threshold used in Figure 2, while a comparison of MDHS and conserved water molecules from the three crystal structures (see Methods section) is shown in Figure 3.

**Figure 4:**
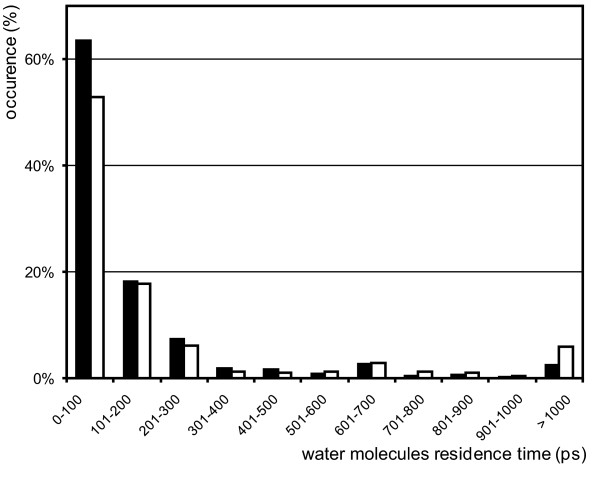
**Distribution profiles of water residence times**. Distribution profiles of water residence times calculated from the MD simulations based on the X-ray (filled bars) [PDB:1C8C] and NMR (open bars) [PDB:1JIC] structures.

**Figure 5:**
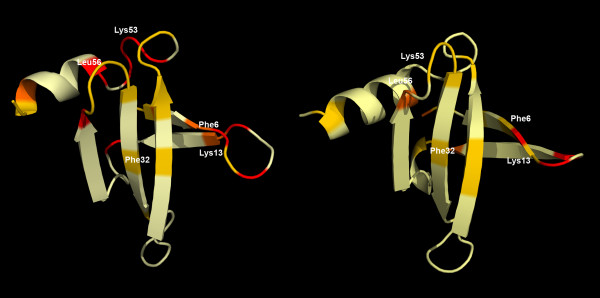
**Residence times of water molecules**. Average residence times of water molecules on the Sso7d surface derived from the MD simulations based on NMR (left) [PDB:1JIC] and X-ray (right) [PDB:1C8C] structures. Pale yellow, yellow, orange and red indicate short (τ < 100 ps), intermediate (100≤τ < 300 ps), long (300≤τ < 1000 ps) and very long (τ ≥ 1000 ps) residence times respectively.

The average residence time (τ) for the water molecules was computed for both MD trajectories according to the method described by De Simone and coworkers [1]. A similar distribution of the residence times between the two was apparent (see Figure 4), except in the range from 1 ns onward; this result will be discussed later. Significantly for the comparison, the monotonic decrease was interrupted in both cases in the 600-700 ps range. The same distributions were used to choose cut-off values for clustering the times into short (τ < 100 ps), intermediate (100≤τ < 300 ps), long (300≤τ < 1000 ps) and very long (τ≥1000 ps) ranges based on their relative populations. This clustering was used to map the obtained τ values onto the Sso7d structure (Figure 5). As observed for the Sso7d MDHS, the τ profiles obtained from MD trajectories using the NMR and X-ray reference structures show significant similarities, although some discrepancies were apparent. In particular, the second half of the segment in the first β-sheet spanning residues Phe6 to Lys13 and the segment (residues 53-56) at the beginning of the C-terminal helix show very longer τ values (in the ≥1000 ps range) when the NMR structure is used for the MD simulation (see Figure 4). This finding can be ascribed to the diminished secondary structure content in these two protein regions during the NMR based MD simulation, see Additional file 1. The incomplete closure of secondary structure elements can lead to a lack of structural constraints in the NMR derived structure that can cause protein backbone amide and carbonyl groups to trap water molecules and form water bridged H-bonds. Despite these potentially misleading artifacts, the general agreement between the two MD simulation results was apparent because all the other minor differences could be ascribed to the expected simulation noise. To further assess the general convergence of the two MD simulations, the τ computation was independently repeated on each half of the two trajectories. The results were clustered as described above and the distribution profiles for the four data sets showed good agreement (see Additional file 2).

The MD results indicate that: (i) the simulation procedures that were used are reliable, because, independently from the reference structure used for the MD run, the Sso7d surface presents similar MDHS distributions and similar τ values; and (ii) because all the water molecules that were conserved in the crystal structures overlap with the MDHS, the hydration site predictions are accurate (see Figure 3).

### ePHOGSY NMR measurements

**Figure 6:**
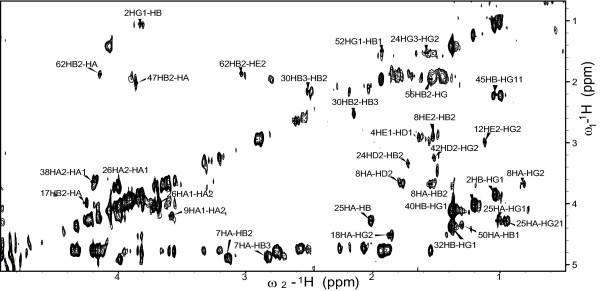
**NMR spectrum of ePHOGSY-TOCSY with NOE**. Contour plot and signal assignment for a region of the 2D ePHOGSY-TOCSY spectrum with NOE.

**Figure 7:**
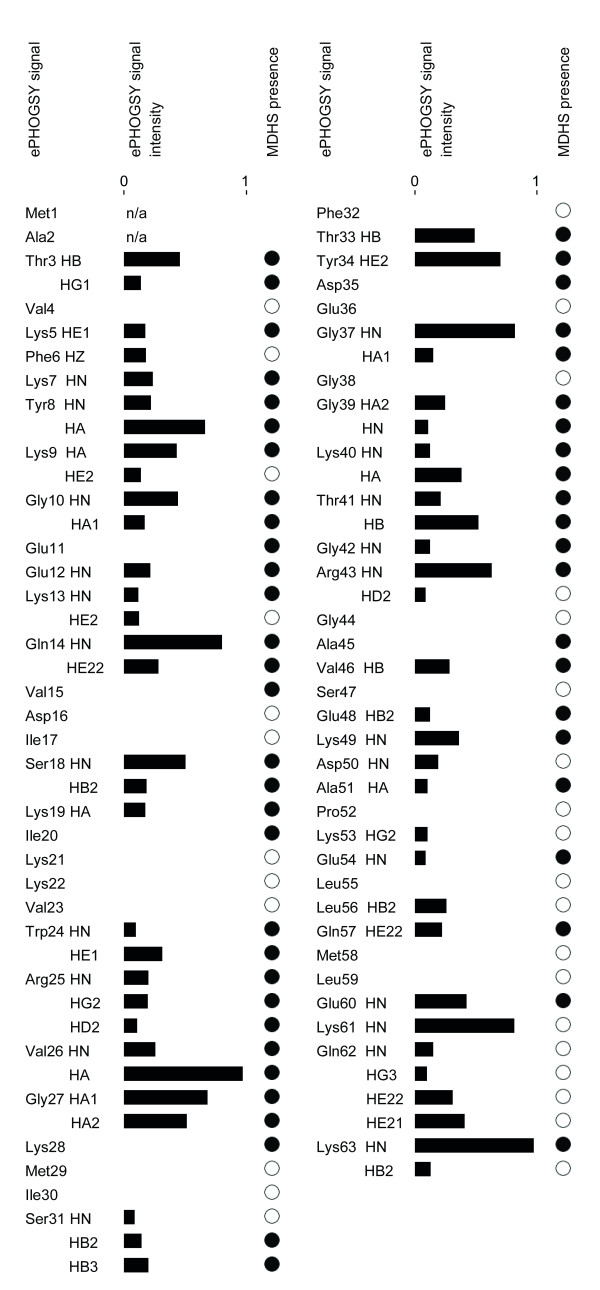
**ePHOGSY signal intensities for NOE_wp _and NOE_(wp)_**. The normalized intensities of the observed ePHOGSY proton signals are shown as filled bars. The presence (filled circle) or the absence (open circle) of a vicinal MDHS (computed for a distance of 0.35 nm from each hydrogen) is shown. For amino acids not exhibiting an ePHOGSY signal (absence of intensity bars), the presence of MDHS was taken as positive if at least one of the hydrogens was within the 0.35 nm cut-off distance.

**Figure 8:**
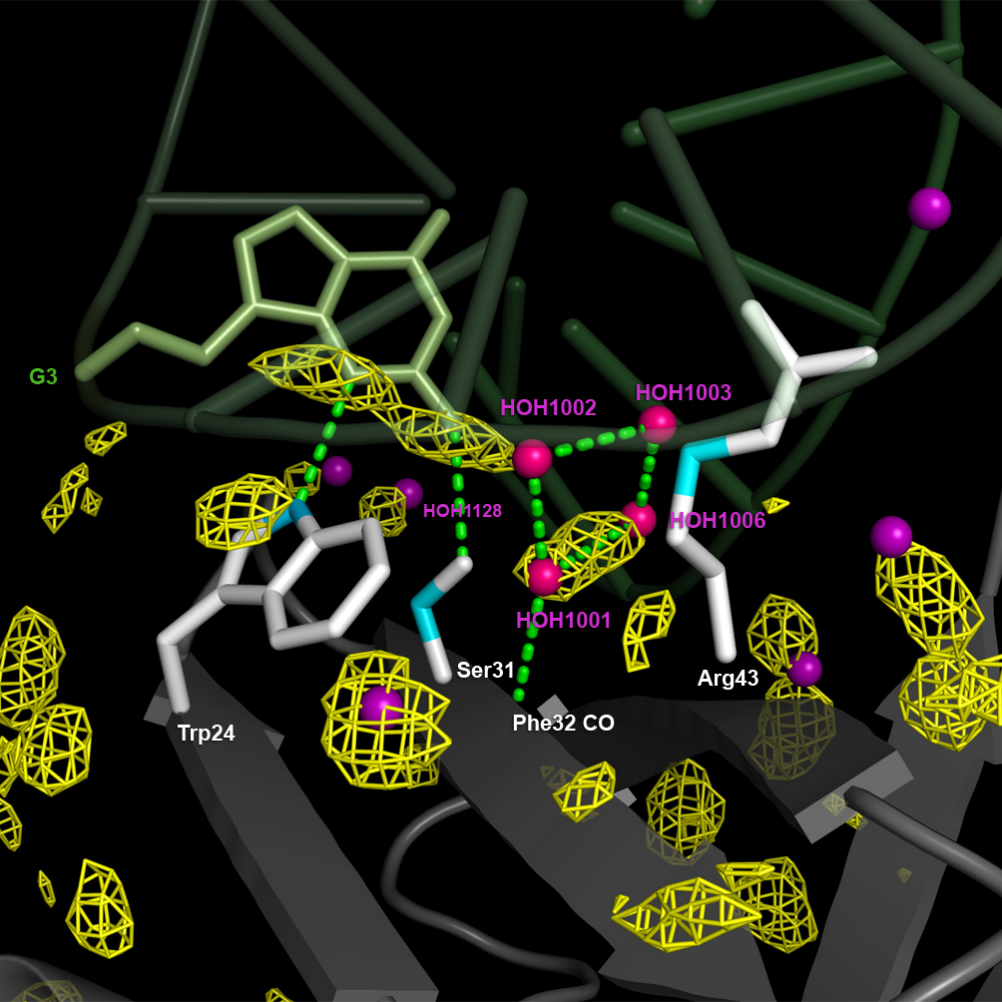
**Detailed view of hydration at the protein/DNA interface**. The Sso7d binding region (grey) with a DNA duplex (green) is shown. Water molecules arranged in a diamond shaped cluster, always found at the Sso7d/Sac7d-DNA binding interface, are colored in red; other water molecules that were found to be conserved in the available crystal structures are shown as purple spheres. The heavy atoms of the amino acid side chains in the presence of ePHOGSY signals are in cyan. The MDHS are shown in yellow; the mesh iso-surfaces were at the 2.8 level.

Intermolecular nuclear Overhauser effects between Sso7d and water were generated using ePHOGSY pulse sequences [10]. In such experiment, signals are obtained only for those protein hydrogens in close contact with the protons of resident water molecules. This is achieved by zeroing the intramolecular NOE signals by dephasing the protein magnetization, while water resonance through selective inversion before the NOE mixing time is preserved, thus yielding only intermolecular water-protein NOE signals. By adding classical NMR building blocks, the basic 1D ePHOGSY experiments can be expanded to multidimensional and multinuclear types such as 2D NOESY, 2D TOCSY and^15^N-HSQC to improve signal dispersion. As shown in Figure 6, in the 2D ePHOGSY-TOCSY spectrum with NOE, many signals were present. 1D and 2D ePHOGSY spectra containing NOE and ROE effects were compared to discriminate signals from direct chemical exchange from signals arising from NOE_wp _or NOE_(wp) _[10]. A total of 61 signals have been ascribed either to NOE_wp _or to NOE_(wp) _(see Figure 7). Distinguishing between NOE_wp_s and NOE_(wp)_s is not an easy task [10,15] because relayed chemical exchange may contribute to the ePHOGSY signal intensities of all the hydrogens that are located within 0.5 nm of surface exposed amino, guanidyl, carboxyl or hydroxyl groups. Based on the Sso7d reference structures used in the present study, all the observed ePHOGSY signals could be attributed to NOE_(wp)_s. Therefore, a quantitative interpretation of the ePHOGSY data in terms of water-protein internuclear distances was not practicable. Different rates of proton exchange between water and protein, if higher than dipolar cross-relaxation, could largely control the intensity of such NOE peaks [30]. It follows that the presence of water molecules at the solvent-Sso7d interface can only be qualitatively inferred by the distribution of ePHOGSY peaks along the protein backbone (see Figure 3). Correlations between the NMR derived hydration sites and the MDHS were quantitatively analyzed by examining the presence of MDHS within 0.35 nm of the hydrogen atoms that showed ePHOGSY signals (see Figure 7). Good agreement between experimental and predicted data was found along the protein backbone. The only exception was for a few protons at the C-terminus of the protein and this finding can be explained by the large fluctuations observed for the end portion of the terminal helix in the MD (see Additional file 3). As mentioned, the MDHS method [4] cannot be used successfully when major protein backbone conformational changes are present. Here it is worth noting that the ePHOGSY signals observed for Ser31 and Arg43 were consistent with the MDHS found in the center of the DNA/protein interface. This finding indicated that the seeds for the formation of the diamond shaped group of water molecules present in the crystal structures of the Sso7d-DNA complexes [25] also existed in the free form of the protein and in solution (see Figure 8).

## Discussion

The hydration dynamics of Sso7d has been investigated through 100 ns MD simulations and ePHOGSY NMR studies. As reported in Figure 2, the MDHS were unevenly distributed on the protein surface, indicating that the Sso7d hydration shell exhibited different local stabilities. A complex network of different contributions that include local flexibility and hydrophobicity in the protein structure determined the modes of molecular traffic of water and other molecules around the protein surface.

By comparing computational and experimental data on the dynamic aspects of Sso7d hydration, several interesting features became apparent. Correlations between the crystallographic water molecules and the MDHS were found only for water molecules that were in conserved positions. Furthermore, even though the signals observed in the Sso7d ePHOGSY spectra could not be unambiguously assigned to NOE_wp _or NOE_(wp)_, the close correlation between the corresponding hydrogens and nearby MDHS was noticeable (Figure 3 and 7). From these findings it can be suggested that signals in ePHOGSY or equivalent spectra are, at least in a qualitative way, diagnostic of the hydration state of the protein. Conversely, the absence of the spectral signals over extended regions of the protein surface strongly supports the presence of a protein hot spot.

The hydration landscape of Sso7d consistently offered by X-ray diffractometry, NMR and MD simulations indicates that, in the protein surface region where binding to DNA and misfolded/unfolded proteins occurs [22,23], a diamond-shaped cluster of four water molecules [25] is anchored to the Phe32-carbonyl located in the center of the binding surface via the water molecule 1001 (see Figure 8). The MDHS close to the Ser31 and Arg43 side chains clearly pointed to the positions of water molecules 1001 and 1006, in agreement with the observed ePHOGSY signals from the β and σ hydrogen atoms of, respectively, the Ser31 and Arg43 side chains in the 4-5 β-strand pair (see Figure 8). Moreover, the Phe32 region in the three stranded β-sheet was the only residue that showing relevant water residence times (see Figure 5).

In the Sso7d-GTGATCGC complex [PDB:1C8C], the backbone amide group of Lys28 and the N3 of G15 from the mismatched T-G base-pair are H-bond bridged via water 1128. A water molecule was found in a similar position for the complex with canonical base pairing [25]. An MDHS, consistently found near the 26-28 protein loop, stabilized the water molecule that interacted with the base-pair (see Figure 8). In the middle of the protein/DNA interaction surface, Trp24-Nε1 and Ser31-Oγ exhibited medium sized ePHOGSY effects that were confirmed by the presence of a nearby MDHS. Comparative analysis of X-ray, NMR and MD results clearly delineated a binding sub-site in this protein region that was partially protected by water molecules that were easily displaced; thus favoring a large variety of interactions. In the DNA-bound structure, the MDHS was replaced by the N_2 _and N_3 _atoms of the G3 aromatic ring, indicating that hydrogen bonding could also be established by the amino acid side chains that support the Sso7d chaperone activity.

It was very interesting to compare the present dynamic hydration profile with our previous investigation of Sso7d surface accessibility using TEMPOL and Gd(III)(DTPA-BMA) paramagnetic probes [24]. The anomalous small paramagnetic perturbations observed for the surface exposed Sso7d P-loop region [24] spanning residues 35-41, can be now interpreted in terms of high solvent density (many MDHS are found in this region) which would have prevented free local diffusion of the two probes (see Figure 3). The MD evidence is strengthened by the presence of four conserved water molecules in the region and by ePHOGSY signals from the backbone amide groups of Gly37, Gly39, Lys40 and Thr41. Furthermore, the lack of an ePHOGSY signal and the MDHS shield for the apical Gly38 residue explains the observed strong paramagnetic attenuation in the presence of Gd(III)(DTPA-BMA).

From a similar analysis, it was apparent how Sso7d loop I, including Tyr8, Lys9, Gly10 and Glu11 residues, was free from MDHS and conserved water molecules (see Figure 3). At the same time, loop I contained the NH group of Lys9 which appeared among the most TEMPOL and Gd(III)(DTPA-BMA) accessible backbone amides [24].

## Conclusions

Combined analyses of MD and NMR results, particularly when data from ePHOGSY spectra and perturbation profiles induced by soluble paramagnetic probes are available, can provide a detailed view of protein surface dynamics. Surface accessibility and hydration of proteins appear strongly coupled, suggesting that the investigation procedure described here represents a new powerful strategy for protein hot spot mapping. It should be noted the dynamic character of the obtained data can offer an unique perspective for delineating transient sites where disruptors of protein-protein interactions can bind.

### Methods

### Data set

Three-dimensional structures, derived from NMR or X-ray experiments, of DNA-binding protein 7d from *Sulfolobus solfataricus*, Sso7d [UniProt:P39476] are available from the Protein Data Bank [29] both in the free form and in complexes with different DNA fragments [PDB:1BF4, PDB:1BNZ, PDB:1C8C, PDB:1JIC, PDB:1SSO]. In the present work, the numbering of the amino acid residues is from the crystal structures, with the first alanine in position 2.

### Conserved water identification

To identify the conserved positions of water molecules in the crystal structures, the method proposed by Knight and coworkers was employed [31]. For each structure, all water molecules within 0.36 nm of a nitrogen, oxygen or sulfur atom of the Sso7d protein were included in the search. After structurally aligning the heavy atoms in the proteins, water molecules from the different structures that fell within a common sphere of 0.2 nm radius and that were found in at least 2 out of 3 structures, were considered as conserved ones.

### Molecular Dynamics

100 ns MD simulations were performed with explicit solvent using the lowest energy NMR structure of Sso7d [PDB:1JIC] [28] and the crystal structure of a Sso7d-DNA complex [PDB:1C8C] as the starting structures after removal of DNA and water molecules. The GROMACS package [32] with the AMBER force fields [33] were used for the MD run of solvated structure in a cubic box of equilibrated TIP3P water molecules [34]. The initial shortest distance between the protein and the box boundaries was set to 1.0 nm and chloride ions were added to achieve global electric neutrality. Afterwards, the energy of the system was minimized with 900 steps of conjugate gradients. To achieve good equilibration prior to the production MD run, the system was subjected to a short (20 ps) MD run during which the atoms of the macromolecule were restrained to their position and only the solvent were allowed to move. The protein-water system was simulated in the NPT ensemble at constant temperature (300 K) and pressure (1 atm); a weak coupling to external heat and pressure baths was applied (relaxation times were 0.1 ps and 0.5 ps, respectively). All covalent bonds were constrained using the LINCS algorithm and non-bonded interactions were computed using the PME method [35] with a grid spacing of 0.12 nm for electrostatic contribution and with a 0.9 nm cut-off for the van der Waals contribution. An integration time step of 2 fs was used and trajectory snapshots were saved every 0.2 ps.

Because the backbone RMSD leveled off after an equilibration period of 100 ps, the subsequent analysis of the MD trajectories was carried out from 100 ps onward.

### Water density function and residence time calculations

Solvent density maps, with maxima defined as molecular dynamics hydration sites (MDHS) [1,4], were calculated from the atomic coordinates of the explicit waters in the simulation. The space surrounding the protein was divided into two shells: the first extended to a distance of 0.6 nm from the protein surface and included the protein hydration sites; the second represented the bulk solvent and extended from 0.6 nm to 0.8 nm from the protein surface. The water positions were computed in a 3D grid (step-size 0.05 nm). For each frame, the protein was superimposed onto a reference structure to eliminate the effects of translations and rotations. A 3D iso-contour plot of the resulting water density was obtained using the PyMOL software package (http://www.pymol.org), see Figure 2. Solvent density map correlation was carried out using MapMan software [36]. The average residence times for the water molecules were computed for both MD trajectories according to the method described in [1]. A residence times profile was obtained by binning values into 100 ps bins for the range 0-1000 ps and in a single bin for 1000 ps onward.

### Sample preparation

NMR samples were prepared by dissolving Sso7d in H2O/D2O (90:10 v/v) to make 1.2 mM protein solutions which were adjusted to pH 4.5 by the addition of small amounts of HCl or KOH. Procedures for protein expression and purification have been reported elsewhere [37].

### NMR measurements

NMR measurements were carried out at 300 K using a Bruker DRX 600 spectrometer to reproduce the experimental conditions of the original structural studies [28]. Data processing and spectral analysis were performed using the, XWinNMR (Bruker BioSpin GmbH, Germany) and Sparky [38] software respectively. 1D ePHOGSY spectra with NOE and ROE were obtained by running 1,024 scans over 8,192 data points and 2D ePHOGSY-TOCSY spectra with NOE (see Figure 6) were obtained using 256 increments and 320 scans over 2,048 data points. In all the ePHOGSY experiments, a spectral width of 8,096 Hz was used, the mixing time to build up the intermolecular Overhauser effects was 202 ms, and the TOCSY mixing time was 75 ms. The water-selective 180° Gaussian pulse in all the ePHOGSY sequences was achieved according to Dalvit [10] with a duration of 25 ms. Water suppression was achieved following the scheme of Hwang [39]. Observed chemical shifts were congruent with those from BioMagResBank (http://www.bmrb.wisc.edu) for the backbone^1^H and^15^N resonances for Sso7d [BMRB entry:5909].

## Abbreviations

ePHOGSY: enhanced Protein Hydration Observed through Gradient SpectroscopY; HSQC: Heteronuclear Single Quantum Coherence; MD: Molecular Dynamics; NMR: Nuclear Magnetic Resonance; NOE: nuclear Overhauser effect; RMSD: root mean square deviation; ROE: rotating-frame Overhauser effect; TOCSY: Total Correlation SpectroscopY.

## Authors' contributions

AB designed the experiments, carried out the NMR and MD work, analyzed the data and prepared the manuscript. OS and SC carried out the bioinformatics analyses. RC and IA supervised the NMR work. PF carried out the expression and purification of^15^N-enriched Sso7d. AG supervised the biochemical aspects. NN supervised all steps of the project and drafted the manuscript. All authors read and approved the final manuscript.

## Supplementary Material

Additional file 1 **Analysis of Secondary structures along the MD trajectories**. Diagram of the secondary structure content along the MD trajectories based on X-ray and NMR derived structures of Sso7d. Click here for file

Additional file 2 **Distribution profiles of water residence times**. Plots of the distribution of water residence times as calculated for each half of the MD trajectories starting from X-ray and NMR derived structures of Sso7d. Click here for file

Additional file 3 **Plot of the root mean square fluctuations of backbone atoms along the MD trajectories**. Root mean square fluctuations of backbone atoms, averaged over residues, of X-ray and NMR derived structures of Sso7d along the respective MD simulation trajectory. Click here for file
