# Supplementary material for: Hydration studies on the archaeal protein Sso7d using NMR measurements and MD simulations
Source: BMC Struct Biol. 2011 Oct 21;11:44. doi: 10.1186/1472-6807-11-44 (PMC3207888; doi:10.1186/1472-6807-11-44)
Supplement: Additional file 1 — Analysis of Secondary structures along the MD trajectories. Diagram of the secondary structure content along the MD trajectories based on X-ray and NMR derived structures of Sso7d. [file 1472-6807-11-44-S1.DOC]

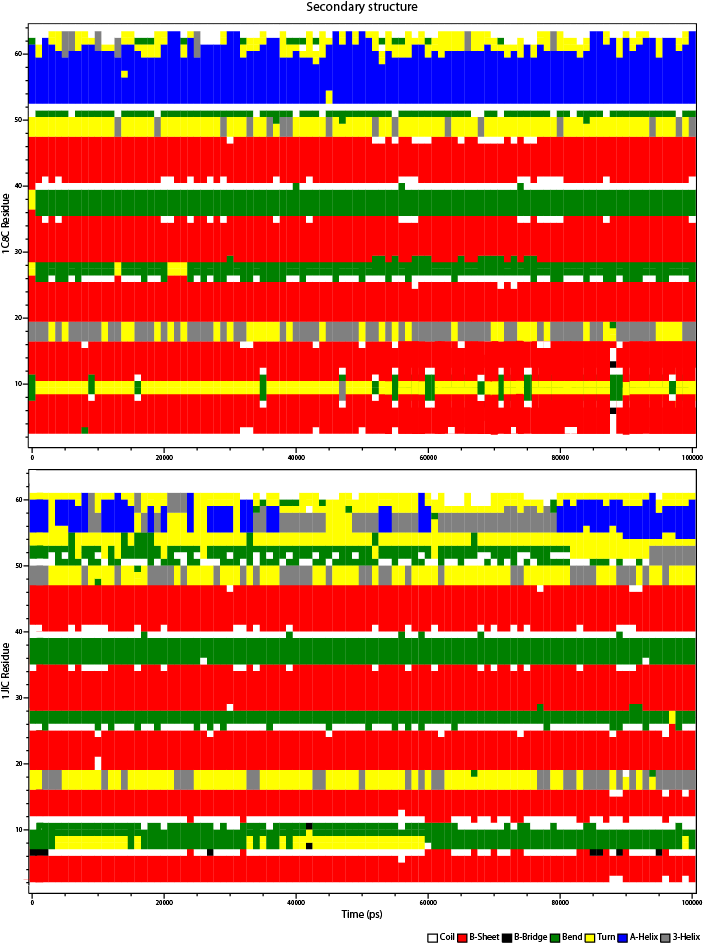


Analysis of Secondary structures along the MD trajectories based on X-ray (PDBID:1C8C, top) and NMR (PDBID:1JIC, bottom) Sso7d structure. Differences in the segments spanning residues 6-13 and 53-56 are apparent.
