# Supplementary material for: Hydration studies on the archaeal protein Sso7d using NMR measurements and MD simulations
Source: BMC Struct Biol. 2011 Oct 21;11:44. doi: 10.1186/1472-6807-11-44 (PMC3207888; doi:10.1186/1472-6807-11-44)
Supplement: Additional file 2 — Distribution profiles of water residence times. Plots of the distribution of water residence times as calculated for each half of the MD trajectories starting from X-ray and NMR derived structures of Sso7d. [file 1472-6807-11-44-S2.DOC]

0-50 ns

51-100 ns

1JIC

1C8C

Distribution profiles of water residence times as calculated for each half (left: 0-50 ns, right: 51-100ns) of the MD trajectories starting from X-ray (PDBID:1C8C, top) and NMR (PDBID:1JIC) structures.
