# Supplementary material for: Hydration studies on the archaeal protein Sso7d using NMR measurements and MD simulations
Source: BMC Struct Biol. 2011 Oct 21;11:44. doi: 10.1186/1472-6807-11-44 (PMC3207888; doi:10.1186/1472-6807-11-44)
Supplement: Additional file 3 — Plot of the root mean square fluctuations of backbone atoms along the MD trajectories. Root mean square fluctuations of backbone atoms, averaged over residues, of X-ray and NMR derived structures of Sso7d along the respective MD simulation trajectory. [file 1472-6807-11-44-S3.DOC]

Root mean square fluctuations of backbone atoms, averaged over residues, of X-ray (PDB:1C8C, blue) and NMR (PDB:1JIC, red) Sso7d derived structures along the respective MD simulation trajectory.
